# Australian Psychologists Experiences with Digital Mental Health: a Qualitative Investigation

**DOI:** 10.1007/s41347-022-00271-5

**Published:** 2022-08-16

**Authors:** Stephanie Scott, Vikki Knott, Amy L. Finlay-Jones, Vincent O. Mancini

**Affiliations:** 1 Discipline of Psychological Sciences, Australian College of Applied Psychology (ACAP), Sydney, NSW Australia; 2 School of Population Health, Curtin University, Perth, WA Australia; 3 Telethon Kids Institute, Perth, WA Australia; 4 UWA Medical School, Division of Paediatrics, University of Western Australia, Perth, WA Australia

**Keywords:** Digital mental health, Implementation, Thematic analysis, Routine practice

## Abstract

Digital mental health is changing the landscape of service delivery by addressing challenges associated with traditional therapy. However, practitioners’ use of these resources remains underexamined. This study explored psychologists’ attitudes and experiences with digital mental health intervention. Taking a qualitative exploratory approach via thematic analysis, the study sought to answer the following research questions: (i) *How do psychologists perceive digital mental health?* and (ii*) What is their experience using digital mental health as part of routine practice?* Ten practising psychologists participated in online semi-structured interviews (approximately 50 min), with interviews then transcribed verbatim. Interview data were analysed according to the six-phase approach to thematic analysis proposed by Braun and Clarke. Three themes were identified: (1) attitudes towards digital mental health; (2) use within routine practice; and (3) perspectives on an effective model for implementation. Practitioners play a major role in the design and delivery of digital mental health services. Barriers and facilitators at the practitioner-level (e.g. knowledge and competence with tools, perceptions on the utility of digital interventions) and the service-level (e.g. government support for digital health) should be considered in the future design of digital mental health resources and service delivery.

**Supplementary Information:**

The online version contains supplementary material available at 10.1007/s41347-022-00271-5.

## Background

The digital delivery of mental health services has provided an alternative, novel and effective strategy compared to services delivered in a traditional face-to-face setting (Sturk et al., 2019). The rapid uptake of digital mental health (also termed *e-mental health*) in the past two decades has transformed the landscape of service delivery by helping to overcome issues relating to accessibility, time constraints, stigma and cost (Carolan et al., 2017; Richards & Richardson, 2012). This has also led to the implementation of government-led strategies to support the ongoing development and deployment of these resources to improve access and reduce the burden on the existing healthcare system (Reynolds et al., 2015). Critically, growing demand for mental health services in Australia—and worldwide—is impossible to meet without leveraging digital technology (Aboujaoude et al., 2020). This has been made especially salient during the COVID-19 pandemic where access to traditional healthcare was impeded (Molfenter et al., 2021; Ojha & Syed, 2020).

*Digital mental health* is defined as internet-based and/or digital technologies (e.g. smartphone applications) that are used to provide therapeutic content directly to consumers, with or without involvement from a trained mental health practitioner (Batterham et al., 2015). This definition differentiates digital mental health from ‘*telehealth*’—which refers to the delivery of traditional services using digital technology. To exemplify, a person using a smartphone application to help challenge unhelpful cognitions would be classified as a form of digital mental health, whilst a person having a consultation with their therapist via teleconferencing software would be considered a form of telehealth. As the definitions for both terms include the digitally mediated delivery of content to manage mental health, it is perhaps unsurprising to see previous research using these terms interchangeably, or under a similar term such as *e-mental health*. However, the distinction between digital mental health from telehealth is an important one.

The increased demand for mental health practitioners (e.g. psychologists, counsellors, social workers)—especially in response to the COVID-19 pandemic—continues to exceed available supply of suitably trained professionals (Balcombe & De Leo, 2021). Thus, whilst telehealth has proven an effective method of connecting people to mental health professionals digitally, it remains constrained by the availability of professionals to provide these services (Ojha & Syed, 2020). Digital mental health, however, has a demonstrated potential to overcome these constraints as content delivery does not have to be delivered by a person. Moreover, digital mental health can also be used to supplement, and even enhance, telehealth or traditional face-to-face interventions that can help to alleviate some of the demand for mental health practitioners from consumers—referred to as a *blended* form of treatment (Titzler et al., 2018).

The blended approach to the delivery of mental health services is particularly relevant to healthcare professionals tasked with the provision of mental health intervention. However, blended modalities have been less-frequently researched compared to purely digital mental health interventions (Titzler et al., 2018). One systematic review of 44 studies suggested that a blended approach can save practitioners time, increase effectiveness of F2F treatment, improve client adherence and help to prevent relapse (Erbe et al., 2017). Another study interviewing 11 European psychologists found that when compared to traditional face-to-face delivery, blended treatment fostered increased client engagement and added flexibility and choice to the therapy process (Cerga-Pashoja et al., 2020). Blended intervention approaches may equip mental health providers with greater scope to work with their clients whilst reducing the burden of care placed on the individual healthcare provided. One common example is the integration of digital *modules* that can be completed between face-to-face sessions, helping to maximise the use of valuable interpersonal time between clients and their practitioners (Cerga-Pashoja et al., 2020). However, existing studies investigating the blended delivery of mental health interventions have mostly done so using a structured approach (e.g. as part of a controlled trial). Currently, it remains unclear whether these prescriptive approaches to blended delivery are feasible in routine practice where client presentations, available resources, workplaces and practitioners vary substantially. Understanding how practitioners are *actually* incorporating digital mental health into their practice can provide nuanced insights that can inform more effective strategies to improve uptake and serve as guide for future research protocols.

The effectiveness of digital mental health services has become difficult to refute. Following the release of early online cognitive behavioural therapy (CBT) program in 2001, there has been a proliferation of digitally mental health services with demonstrated efficacy for many types of mental health conditions and personal contexts (Baus & Bouchard, 2014; Hilvert-Bruce et al., 2012; Orman & O’Dea, 2018; Park et al., 2019; Tait et al., 2019; Titov et al., 2016; Werner-Seidler et al., 2019). Much of the current digital mental health content continues to draw upon the principles of cognitive behavioural therapy (CBT). More than 200 randomised control trials (RCTs) have been published on digital CBT programs, and most have indicated that iCBT is as clinically effective a treatment as conventional face-to-face treatment (Andersson & Titov, 2014). However, content derived from other types of therapy, such as interpersonal therapy, psychodynamic therapy, structured writing techniques and eye-movement desensitisation, has also proven effective for many types of mental health concerns (Donker et al., 2013; Johansson et al., 2013; Lange et al., 2003; Reynolds et al., 2015; Spence et al., 2013). Therefore, a contemporary perspective in support of digital mental health might suggest that almost all mental health services provided in a traditional face-to-face setting could also be delivered digitally to a similar degree of effectiveness—as has magnified by the COVID-19 pandemic. However, there remain several barriers to digital mental health services reaching its full capability (Aboujaoude, 2018; Orman & O’Dea, 2018).

Barriers that restrict the access or effectiveness of digital mental health services exist across multiple levels. For example, a lagging infrastructure and skill base has impacted the development and delivery of digital mental health services at the broader level despite growing government recognition of its merits (Balcombe & De Leo, 2021; Reynolds et al., 2015). At the individual level, factors such as access to technology and technological literacy and beliefs about the efficacy of digital mental health services influence decisions to engage in these types of services. For blended modes of delivery, mental health practitioners act as important mediators of their client’s engagement with digital mental health services. The delivery of digital mental health within routine practice is dependent on practitioners’ attitudes and engagement with these resources (LaMonica et al., 2019; Sinclair et al., 2013). Several studies have applied behavioural change and innovation adoption theories to the implementation of digital mental health (Feijt et al., 2018; Hennemann, 2017; Lovejoy, 2009). However, at times, these studies have not considered the influence of individual differences in practitioners’ willingness and experience to explore and use digital mental health tools (Feijt et al., 2018). To address this, Feijt et al. (2018) proposed a new implementation theory—*Levels of Adoption of eMental Health (LAMH)* model. This model suggests that there are five levels of digital mental health adoption that practitioners can experience ranging from zero-use and high scepticism about digital mental health, through to a high level of personal interest and positive outlook bout its efficacy. The LAMH model also offers pragmatic regarding potential drivers, barriers and requirements for change at each level (Feijt et al., 2018). To encourage uptake and use of digital mental health within routine practice, different strategies may be required depending on a practitioner’s level of adoption and engagement.

In Australia, there has been an increased emphasis towards a *stepped care* model of healthcare delivery, with low-intensity or non-specialist services acting as a ‘first-step’ of care, with treatment increasing in intensity or specialisation as needs become more complex (Gunn et al., 2018). Current government policy recommends digital mental health as a first step in treatment for subthreshold and mild presentations of common mood disorders (Department of Health and Aged Care, 2021; Gunn et al., 2018). Where purely digital mental health services have been criticised for their inability to offer ongoing monitoring of client progress and alternative sources of support if a prescribed digital mental health intervention is not meeting the client’s needs (Rosenberg et al., 2020), blended models of delivery may be able to address these criticisms. However, little is known about how allied health professionals are using digital mental health resources within Australian practices (Bagarić & Jokić‐Begić, 2020; Cerga-Pashoja et al., 2020; Dijksman et al., 2017; Reynolds et al., 2015). To address this gap, Reynolds et al. (2015) proposed five main ways that practitioners can integrate digital mental health within their practice: (1) promotion, (2) case management, (3) coaching, (4) symptom-focused treatment and (5) comprehensive therapy. Emerging evidence supports suggestion that Australian practitioners are integrating digital mental health with considerable variation, ranging from recommending websites through to formally incorporating programs, though further research is necessary (Reynolds et al., 2015).

The current qualitative research focuses on the perspective of psychologists in Australia. It aims to understand their lived experience with digital mental health by exploring their attitudes and use of these resources as part of their current practice. Applying a qualitative semi-structured interview method, this study addresses the following research questions: *How do psychologists in Australia perceive digital mental health; and what is their experience using digital mental health as part of routine practice?*

## Method

### Participants

Ten psychologists registered to practice in Australia by the Australian Health Practitioner Regulation Agency (AHPRA) were recruited using a combination of convenience and snowball sampling. Most participants were female (*n* = 9) and aged between 28 and 60 years (*M* = 37.4 years, SD = 6.8 years). All participants were based in Australia and located in Brisbane (*n* = 4), Sydney (*n* = 2), Melbourne (*n* = 2) and Perth (*n* = 2). They held varying levels of professional psychology experience (less than 1 year to 35 years; *M* = 9.4 years, 9.5 years), held general registration as psychologists or were endorsed in clinical psychology, and worked across a range of settings (i.e. government agencies, hospitals, not-for-profits and private practice). Participants also had experience with a variety of populations including children, adolescents and adults with mild, moderate and severe conditions. Participants were all actively practising psychology and reported using a range of therapeutic models including CBT (Beck, 1976), interpersonal therapy, family therapy, play therapy, positive psychology and psychoanalytic therapy.

### Materials

A semi-structured interview schedule was developed for the purposes of the current study. Ten open-ended questions were developed by two of the study authors (SS and VM), with prompts to encourage a breadth and depth in responses. Questions included in the schedule were inspired on constructs relevant to theory on adoption and implementation (Feijt et al., 2018; Hennemann, 2017; Lovejoy, 2009). Questions employed a focus on awareness, attitudes and experiences. Examples of interview questions were as follows: ‘*How would you describe digital mental health*?’, ‘*From your perspective, what may be some of the benefits associated with digital mental health*?’ and ‘*What has been your experience using digital mental health resources*?’ Other questions and prompts sought to understand participants experience as a psychologist and challenges they face as a practitioner (see Supplementary Materials for the full interview schedule). This interview schedule was piloted on two additional registered psychologists and refined based on feedback. An advantage of the semi-structured interview schedule was the opportunity to ask all participants the same core set of questions, but with sufficient flexibility to explore topics in further detail as raised by participants.

### Procedure

This project was approved by a Human Research and Ethics Committee at the lead author’s institution of research (Approval Number: 602090620). Participants were recruited via convenience and snowball sampling through the Australian Psychological Society (APS) website and social media. Participants were required to be a registered psychologist and based in Australia. Recruitment strategies included posts on social media forums including Facebook and LinkedIn. Details of the study were also posted on the Australian Psychological Society’s (APS) website and sent to some of the psychologists that were registered on the APS directory.

Prospective participants were directed to the study’s Facebook page and invited to contact the researcher via a university email to express their interest in the study. As a gesture of appreciation for their time, participants received a $40 voucher upon completion of the interview. All interviews (*n* = 10) were conducted via video using Zoom software to maximise convenience for participants to enable greater geographic diversity in sampling across Australia. These interviews were conducted in the second half of 2020, thus capturing recent changes to standard practice in response to the COVID-19 pandemic. Interviews lasted between 44 and 65 min. Interviews were recorded and transcribed verbatim. The transcripts were sent to each participant for member-checking and approval. At this point, participants were also emailed the gift voucher. Pseudonyms were then applied to transcripts to preserve confidentiality and anonymity. De-identified transcripts were thematically analysed using NVivo software.

### Data Analysis

Interview data were analysed according to the six-phase approach to thematic analysis proposed by Braun and Clarke (2006). Based on the research aim and its exploratory nature, thematic analysis was deemed to be the most appropriate method of analysis. This is because it allows the researcher to see and make sense of collective and shared meanings and experiences (Braun & Clarke, 2006). Throughout analysis, an inductive interpretive approach was applied. This approach to analysis is consistent with an epistemological perspective, which aims to capture the phenomenological experience of participants’ perception and experience with digital mental health. For phase one of the thematic analysis process (Braun & Clarke, 2006), the lead researcher (SS) familiarised herself with the data by reading through the transcripts several times. For phase two and three, initial codes were generated (*n* = 40) and further grouped into draft themes (*n* = 7). As part of the coding process, direct quotes and participant pseudonyms were grouped beside the related code in a spreadsheet. These codes were also presented to the senior study author (VM) and used to generate the themes that were refined by each of the study authors, and agreed upon by the remaining study authors. This provided a clear illustration of each code and an indication of the frequency of each code. Whilst frequency is not necessarily a measure of significance, it offers a sense of the extent to which an experience was common across responses. This can suggest that a construct is more broadly shared. To improve trustworthiness (Sanjari et al., 2014), an indication of the number of participants who addressed each topic has been provided throughout the “Findings” section.

## Findings

The dominant themes that were identified comprised the following: (1) attitudes towards digital mental health; (2) use of digital mental health within routine practice; and (3) perspectives on an effective model for implementation. Within the themes, several subthemes were also identified (see Table 1).

**Table 1:** Themes, subthemes and supporting quotations generated using thematic analysis

Theme	Subthemes	Example quotations
Attitudes towards digital mental health	Psychologists’ definition of digital mental health Attitudes are dynamic and changing Perceived benefits and disadvantages of digital mental health	*‘Something that involves technology in some form.’* *‘My perspective has really changed now. I kind of see it now as a really good option in terms of accessibility.’* *‘Benefits are really about accessibility and ease.’*
Use of digital mental health within routine practice	How digital mental health is used Finding and evaluating digital mental health Barriers to use of digital mental health	*‘I don’t need to (use programs)—I sort of think you do one or the other particularly in eating disorders.’* *‘If it’s a really complicated kind of system to use I probably wouldn’t choose something like that.’* *‘Knowing what’s out there, and what I could kind of recommend would be helpful.’*
Model for implementation	The model for roll-out	*‘The client just feels like they are just being ping ponged around everywhere and that they don’t fit and no one can help them.’*

This table was generated on data gathered during semi-structured interviews (*n* = 10) with psychologists in Australia. It was analysed by two researchers using thematic analysis

### Theme 1: Attitudes Towards Digital Mental Health

#### Subtheme: Psychologists’ Definition of Digital Mental Health

Overall, digital mental health was considered a broad term that was interchangeable with e-mental health. Described by one participant as ‘the use of technology to help improve someone’s mental health’, and another as ‘methods and approaches other than just face-to-face’, participants viewed digital mental health as a comprehensive list of resources suitable for many contexts. Interestingly, although research tends to differentiate digital mental health from telehealth (Batterham et al., 2015), most participants (*n* = 9) considered them to be part components of a broader approach to digitally mediated mental health interventions. As summarised by one participant:

*When I think digital mental health, I think more of like, video sessions. But I know there’s lots of other ways of that. I tend to see it more as that, but I realise it encompasses a lot more than that.*

All participants (*n* = 10) considered ‘apps’, ‘websites’ and ‘online programs’ to be digital mental health resources. Emerging technologies like ‘VR’ and ‘biofeedback’ were also mentioned by participants (*n* = 2) that had direct experience using them. There was discrepancy as to whether online tools to conduct ‘initial assessments’ constituted digital mental health resources with some participants simply seeing them as a strategy to streamline their workflow.

#### Subtheme: Attitudes Are Dynamic and Changing

The implications and experiences during COVID-19 demonstrated this, particularly with telehealth. Participants who were using video telehealth (*n* = 4) prior to COVID-19 reported that the increased use of it was ‘*not a big-deal*’ for them and they already thought it was ‘*fantastic*’. They were excited about the ‘*accelerated trajectory*’ of telehealth and felt that the current level of acceptability for telehealth would have been at least ‘*three years off*’ prior to COVID-19. Participants using video telehealth for the first time (*n* = 6) during COVID-19 reported that their attitudes towards it ‘*really changed*’ (Krissy). Several participants (*n* = 4) found it ‘*challenging*’ and ‘*difficult*’ to deliver all sessions via telehealth. However, they all recognised that it is an important option to offer as it can deliver support in a more ‘convenient’ way that meets ‘*different client needs*’. As described by one participant:

*I think, you know, maybe four or five months ago, I would have been like: ‘No, I don’t want to do that’. Honestly, I think I was under the belief that it would take away from therapy, like doing it online. My perspective has really changed now. I kind of see it now as a really good option in terms of accessibility and, you know, being able to provide support to people that may not have been able to, you know, access it in the traditional sense.*

More broadly, participants noted that the perception of digital mental health is ‘definitely changing’ and moving in a more ‘positive direction’. However, a division of attitudes was evident:

There’s those of us that think it’s fantastic; we’re already using it as part of our treatment versus those that think it’s like, you know—the work of the devil.

According to half of the participants (*n* = 5), practitioner resistance to digital mental health can be due to lack of ‘*experience with it*’ (*n* = 2), ‘*fear*’ (*n* = 4) and ‘*rigidity*’ (*n* = 3). For example:

*There’s some Luddites out there that don’t trust anything and refuse to use any digital mental health because they see it as an affront, a personal affront. And it’s like: ‘No, you couldn’t possibly get benefits from doing an app or doing something online. Therapy is between us, in this room and it’s about my skill’ and all the rest of it.*

#### Subtheme: Perceived Benefits and Disadvantages

All participants (*n* = 10) perceived accessibility to be an overarching benefit for both digital mental health and telehealth. Participants reported that its *far-reaching* (*n* = 5), ‘*flexible*’ (*n* = 7) nature helped to break down mental and physical barriers to access. For example, it provides opportunities for rural and remote (*n* = 3) populations, people with disorders like social anxiety (*n* = 5) or phobias (*n* = 2), or those with physical disabilities (*n* = 2) to access mental health support. The opportunity to meet different client preferences (*n* = 8) was perceived to be another key benefit of digital mental health and telehealth. Participants noted that certain clients had a preference to access support via digital means (*n* = 3).

One participant who worked within the telehealth space, emphasised that clients have different communication preferences, and a benefit of telehealth and digital mental health is that it allows ‘*flexibility for how people want to connect*’ and access support—thus enabling previously treatment-resistant individuals to engage with services.

However, participants perceived key disadvantages of both digital mental health and telehealth to be ‘*increased risk*’ (*n* = 8), ‘*lack of security and confidentiality*’ (*n* = 6), ‘*poor client fit*’ (*n* = 7) and ‘*technological limitations*’ (*n* = 4). As stated by one participant:

I feel like now people just assume everyone has the technology and knows how to use it, but some people don’t. Some people don’t know how to use it. And that’s a big gap.

Another participant noted issues with risk: *‘With anything whether you’re talking about anxiety, depression, suicidal ideation, trauma, whatever it is the less time you have with an actual clinician, potentially the more the risk goes. That’s just how it is, you know, that’s the downside.*’

For digital mental health, there was the added concern around the *‘lack of personalisation’* (*n* = 7) and ‘*lack of the human connection’* (*n* = 6). As a result, most participants viewed the standalone delivery of digital mental health to be inferior to face-to-face treatment as it lacked human contact. This sentiment is best summarised by the following two quotes from different participants:

*[You] have to have a platform that’s humanistic [sic]. You can’t lose sight of that human aspect of mental health - you don’t want it to become so automated that you that they don’t know who they’re talking to when they’re going through a process that is very sensitive. Some of those more automated wellness apps and programs that don’t take into account the human variable.*

*I feel like there is such a big part of seeing a psychologist or a therapist is the connection and the relationship and feeling comfortable. I wonder if that might not be as available if people are solely relying on digital mental health.*

### Theme 2: Use of Digital Mental Health Within Routine Practice

#### Subtheme: How Digital Mental Health is Used

Participants’ level of engagement with digital mental health varied, from ‘*infrequent*’ or ‘*informal*’ use through to a much more ‘*integrated*’ approach. Those with lower levels of engagement (*n* = 5) reported limited use of digital mental health. Most had a relatively positive perception of digital mental health but disclosed fairly limited knowledge regarding the services that are available. Most commonly, they reported using ‘apps’ for mindfulness and referring to ‘websites’ for additional psychoeducation or support services.

Inversely, some participants had several years of experience using digital mental health. For these psychologists, they reported having a ‘toolkit’ of digital mental health resources, mostly comprising of ‘apps’. These participants use apps for mindfulness, safety planning, emotional regulation and event scheduling, as well as for specific conditions. These participants also had greater awareness of online programs. Most did not choose to integrate it into their treatment protocol but would sometimes refer a client to them if they could no longer come to face-to-face treatment (*n* = 2) as part of a stepped care or aftercare program. Unlike ‘apps’, participants often did not feel as though online programs were as easy to incorporate into their treatment protocol. It was usually perceived as a dichotomy. Concerns about ease of use, consistency with current treatment and additional cost prevented participants from using or referring clients to online programs more regularly.

All participants (*n* = 10) reported usually refraining from spending too much time using the digital mental health resource within the session. Instead, they prefer to introduce the client to the ‘app’ or resource, develop a strategy for them to use it outside of the session and then discuss their experience in the next session. This process was summarised by one participant:

*I don’t want to spend time in session on it if they can do it outside of session. But if they have trouble with it, then I’ll do it in session with them. They are usually quite happy to go away and practice that and come back and talk about it.*

All participants (*n* = 10) viewed digital mental health as a strategy to ‘*extend therapy*’ and provide extra support to clients. Particularly with ‘apps’, participants felt like they were a way to upskill clients, give them a sense of independence from the psychologist and provide them with strategies and support outside of the therapy room. As one psychologist described it:

*I don’t have a model that clients are going to see me forever. I want people to get better and to get on with their life. And so it’s kind of a bit more independent. It’s like having a therapist in their pocket on the phone.*

#### Subtheme: Finding and Evaluating Digital Mental Health

According to most participants (*n* = 8), finding and assessing digital mental health resources is a manual process that requires initiative and time. Those at the more engaged end of the spectrum may actively seek out and share digital mental health resources, whilst other means of discovery are through colleagues (*n* = 4), press (*n* = 3), social media (*n* = 4) and clients (*n* = 7). When assessing a new resource, there were some common criteria that participants reported considering. Participants all noted that they would not recommend something until they had looked at it first. ‘*Usability*’ (*n* = 5), ‘*target population*’ (*n* = 7), ‘*cost*’ (*n* = 3), ‘*research*’ (*n* = 3) and ‘*consistency with current treatment*’ (*n* = 8) were all important considerations when assessing a resource. Several participants felt that if the resource differed to their therapeutic approach that it would be challenging to navigate. For example, this discrepancy was described by one participant:

*I had a client complete an online OCD program. And for the most part, it was great, but there were certain things that they would bring up that is completely opposite what we’re discussing, and it was really confusing for them. So yeah, it gets tricky.*

The usability and user experience of resources, particularly for ‘apps’ and online programs, was at the forefront of participants’ minds. Whilst research is a consideration, the context in which a digital mental health tool is being used influences the weight that they give these criteria. For example, research becomes more important if the ‘app’ or program’ is being used as a standalone intervention.

*Whether or not the app has empirical support doesn’t concern me so much, when I’m using it as an adjunct. In that situation, the evidence is me - what I’m doing with that person. This is just assisting that. If the app or program was standalone as their only mental health treatment as a low intensity intervention, then of course, you’d need to have some evidence.*

#### Subtheme: Barriers to Use of Digital Mental Health

Despite expressing a desire to use digital mental health more regularly, lack of awareness (*n* = 6), training (*n* = 4) and knowledge about what is available (*n* = 5) are major barriers to use. As summarised by one participant:

*Getting the right resources for what you need is a big challenge and knowing where they are, knowing how to find them, knowing who they are suitable for and knowing what you have to pay.*

There appeared to be willingness to use more digital mental health within routine practice, though the time to thoroughly learn about the specific digital mental health intervention was a barrier to implementation. Moreover, some of the participants described that a lack of training and development opportunities to develop knowledge and competency in the delivery of digital mental health further obstructed their willingness to implement it.

Inversely, participants disclosed several facilitators to the incorporation of digital mental health in their practice. First, participants associated ‘apps’ with better adherence compared to online programs. Second, digital mental health services that offer some degree of flexibility in content (e.g. allowing a practitioner to recommend a client a specific set of relevant content) were deemed to be more suitable for use than pre-developed content that may also include irrelevant information.

### Theme 3: Model for Implementation

Despite holding relatively positive perceptions of digital mental health, participants voiced concerns (*n* = 6) about its roll-out within the Australian healthcare system. Specifically, their primary concern was the government emphasis on a ‘stepped care’ model. Participants were concerned that clients may be funnelled into a self-help support option as a default, which will not always be able to meet the client’s needs. They also reflected on sentiments raised by clients about feeling ‘ping-ponged’ around the health system and expressed particular concern regarding the appropriate triaging of clients to ensure that they receive the services that they need, rather than only those that are most accessible. The participants emphasised the importance of not just deciding based on severity but also on what the client wants. For example:

*I think there needs to be some probably appropriate assessment of someone’s symptom level. But then also what does a person want? Because it’s that human connection. It’s that understanding and that validation of what someone’s going through that’s actually therapeutic*.

According to some participants (*n* = 4), a risk of the at-risk people being funnelled into standalone online programs is that a bad experience may diminish a client’s help-seeking behaviour in the future. The way it is promoted to consumers is important so that people do not feel like they are ‘*being pushed away from face-to-face services*’, but also do not view digital mental health ‘*as a panacea for everything it’s not*’.

## Discussion

The aim of the current study was to identify how Australian psychologists perceive and implement digital mental health as part of routine practice. The formative qualitative work undertaken in the current study identified the key themes shared by 10 Australian psychologists with various areas of expertise and exposure to digital mental health, helping to gain a variety of perceptions about the current landscape of digital mental health implementation, as viewed by mental health practitioners. The results revealed three broad themes discussed, namely psychologists’ attitudes towards digital mental health, the use of digital mental health within routine practice and a proposed model for the wider implementation of digital mental health across Australia.

Though many of the Australian psychologists interviewed in the present study expressed some hesitation regarding risk, client suitability and lack of human contact in digital mental health, all were amenable to using various forms of digital mental health within routine care as part of a blended model of delivery. In line with previous research (Cerga-Pashoja et al., 2020; Erbe et al., 2017), most participants perceived digital mental health as a method to increase accessibility, provide clients with flexibility and choice, extend therapeutic benefits and increase the effectiveness of treatment. Participants felt that by providing clients with digital tools outside of the session, they were able to consolidate their learning more effectively. All participants had at least some experience using digital mental health products; our insights into the perspectives of those without prior experience were limited; thus, support for digital mental health may reflect the well-established relationship between prior experience and acceptability of digital mental health (Gun et al., 2011). Some participants also noted a shift in their attitudes during because of the COVID-19 pandemic beginning during the same year that these interviews were conducted. Though the sentiments expressed about COVID-19 were more closely aligned with telehealth and not digital mental health, participants did acknowledge a greater use of digital mental health as a result. The sudden adoption of telehealth and digital mental health in response to the pandemic may place increased pressure on an already lagging infrastructure and skill base of digital solutions in mental health care, whilst these experiences were beyond the remit of the current study aims, it is important to reflect on how these may have flavoured the interviews conducted (Balcombe & De Leo, 2021). The pandemic has accelerated mental health practitioners’ awareness and experience of digital interventions, yet it remains unclear whether the rapid shift towards the integration of technology has improved, or diminished, the opinions of mental health practitioners in Australia (Balcombe & De Leo, 2021; Ojha & Syed, 2020).

Despite the mostly positive attitudes held by participants in our sample, practitioner resistance remains a barrier to uptake (Batterham et al., 2015; Clough et al., 2019; Knott et al., 2020; LaMonica et al., 2019). Whilst the psychologists in our sample held mostly positive beliefs about digital mental health, they did report that other colleagues in their industry were resistant to the idea of clients accessing digital mental health services, especially those that were self-directed or did not involve clinician-input. However, these perceptions of resistance were mostly attributed to a lack of knowledge about how to implement digital mental health, or how it can act as a companion to existing models of care. As described by the five-level *LAMH* model by Feijt et al. (2018), the current findings support the suggestion that experience mediates acceptability of digital mental health. Importantly, the participants in our study could be classified as having a passive (level 3) to active (level 5) level of engagement—meaning that those with very low levels of adoption were not interviewed directly (but were commented on by those interviewed). As predicted by Feijt et al. (2018), our participants also perceived different barriers and drivers to adoption based on their level of experience with digital mental health. For example, participants with ‘passive’ levels of adoption perceived lack of awareness and knowledge of digital mental health to be their biggest barriers. Conversely, participants with ‘active’ levels of adoption were more likely to fill this knowledge gap by proactively seeking out new digital mental health resources. This also highlights an important implication for the rollout or upskilling of digital mental health knowledge, as psychologists with different levels of adoption will require different strategies to improve uptake. One must not assume that all psychologists have the same level of experience with digital mental health, even in a post-pandemic climate.

Understanding the unique experiences of Australian psychologists currently providing mental health services is of relevance given incoming changes to standard mental health service provision, with the stepped model of care to be implemented as a strategy to reduce burden on an already-strained mental health care system (Department of Health and Aged Care, 2021). The stepped model enables clients with lower-severity conditions to access a less-intensive type of support, with digital mental health services that have little clinician involvement poised to be the primary strategy to meet demand from the public (Orman & O’Dea, 2018). For example, rather than seeing a psychologist or receiving pharmacological intervention, people are referred to a digital mental health program (standalone or guided) as a first step. Those psychologists interviewed in the current study expressed similar concerns reported in a previous study by Rosenberg et al. (2020), with this model making it difficult to redirect clients to alternative supports if the online programs do not provide a good fit and may dissuade clients from further help-seeking.

The psychologists interviewed in this study perceived digital mental health as a positive yet inferior method of mental health intervention when compared to traditional interpersonal approaches (e.g. psychologist-client contact in face-to-face or telehealth modalities). Standalone or clinician-guided delivery of digital mental health was perceived as a good option when access or cost concerns were raised, or when digital mental health was considered to ‘supplement’ traditional approaches. These perceptions are incongruent with prior studies that have shown digital mental health interventions to report similar effectiveness as face-to-face delivery. However, this discrepancy may be unsurprising, as the psychologists interviewed in this study are likely to have placed high value on the therapeutic alliance between clients and practitioners; participants expressed greater acceptance of digital mental health when integrated with face-to-face approaches, rather than as a standalone and guided intervention. Similar sentiment has been reported in previous qualitative and quantitative research as well (Erbe et al., 2017; Kemmeren et al., 2019; Topooco et al., 2017). These factors should be taken into consideration when promoting the stepped care model to health practitioners and the public. It highlights the importance of establishing effective processes for triaging and providing clients with appropriate steps to access other mental health support if online programs are not a good fit.

Prior research has demonstrated that many Australian mental health practitioners are already integrating digital mental health into their routine practice—ranging from recommending websites through to formally incorporating programs in their treatment approaches. However, this is an under-researched area and practitioner use of digital mental health remains poorly understood (Cerga-Pashoja et al., 2020). Whilst the present qualitative finding supports this integration, it provides only a small-scale (*n* = 10) investigation; thus, a larger-scale study of Australian psychologists is recommended. Such research will be able to provide important insight regarding *what* digital mental health services are currently used by Australian psychologists, and *how* these services were identified. A potential barrier to the adoption of digital mental health by Australian psychologists identified in our study was the time required to find and assess digital mental health tools, with many of these tools most frequently introduced to psychologists by their clients. Whilst government-level efforts to develop a repository of digital mental health resources for clinicians have been developed in Australia (e.g. the *Head to Health* platform), participants in the current study did not refer to such platforms as places where digital mental health services were identified. These findings are consistent with a larger study that demonstrated that 81% of healthcare professionals had not heard of the *Head to Health* platform (Sturk et al., 2019). Thus, ongoing effort to build awareness of these resources among practitioners is needed, particularly for those professionals who do not have clients introducing them to different digital mental health platforms. As client acceptability has been demonstrated as integral for use and adherence to digital mental health (Lal & Adair, 2014), the current findings extend this sentiment to clinicians and their role in implementing or introducing digital mental health into their practice.

### Strengths and limitations

The qualitative design used in the current study helps to provide a rich, detailed account about how Australian psychologists are perceiving and using digital mental health as part of their routine practice. Much of the insights gained from the current study would be unlikely to have been ascertained using quantitative methodologies. Though not a pre-requisite for study inclusion, all participants had some degree of experience using digital mental health as part of their treatment, ranging from non-intensive procedures (i.e. referring clients to online resources) to the use of virtual-reality technology and biofeedback. This mix of experiences, coupled with variety in age, years of experience and area of work, helps to increase the transferability of this research—a strength in qualitative research (Willig, 2013). However, it is important to acknowledge that the current findings are derived from in-depth interviews (approximately 45 to 60 min each) with 10 Australian psychologists—the opinions expressed by these 10 psychologists may not be wholeheartedly representative of the broader population whom they represent in this study. The current sample (*n* = 10) is comparable to similar qualitative work undertaken in a European sample (*n* = 11; Cerga-Pashoja et al., 2020). Rather than be considered as definitive in nature, the current findings may be used to formulate a complementary quantitative study recruiting a larger sample. Whilst multiple Australian states were represented in the current study, the inclusion of participants from Australia’s larger cities means that the experiences of participants working in rural and remote areas are not represented in the present study. It is possible that psychologists working in these could conditions are more likely to use digital mental health (as they often service a larger and more geographically disparate population). However, an alternative perspective may be that access to digital mental health is reduced in rural areas. Future research capturing the experiences of rural healthcare professionals is also recommended.

One important consideration for the current study is the timing at which data were collected. Whilst the study aims were developed in early 2020, the COVID-19 pandemic reaching Australia around March 2020 resulted in a complete transformation regarding the provision of healthcare delivery. For mental health practitioners, transition from traditional, in-person delivery of mental health services were replaced with telehealth as part of the country’s rapid response to COVID-19. Thus, completing interviews in the second half of 2020, and shortly after the height of the COVID-19 pandemic, may have inadvertently explored participant’s experiences at a time where the digital delivery of mental health services was a necessity rather than an option. Deliberate attention was given to the construction of the interview questions (focussing on digital mental health rather than telehealth); however, it was perhaps unsurprising that many of the participants reflected upon their telehealth experiences given the transition from their in-person practice to a telehealth format. Our results did not intentionally seek to disentangle the influence of the COVID-19 pandemic on psychologist’s perceptions of digital mental health, though many of the participants did this organically as part of the interview. Nevertheless, specific research seeking to understand how the landscape of mental health provision from psychologists (and allied health professionals) following the pandemic is important. With ongoing restrictions still in place over 2 years after the beginning of the pandemic, it is likely that the landscape of mental health provision may have been permanently altered. Therefore, future research may seek to question how psychologists have adjusted to the increased demands or requirements for telehealth and/or digital mental health services.

## Conclusions

This research provides new insights about Australian psychologists’ attitudes and use of digital mental health within routine practice. As discussed, this has important implications for the future design of digital mental health resources and service delivery. These insights can also be used to develop innovative strategies for uptake within routine care. Future research investigating the effectiveness of implementation strategies designed to improve uptake of digital mental health in clinical practice would be valuable.

## Supplementary Information

Below is the link to the electronic supplementary material.

Supplementary file1 (DOCX 27 KB)
