# Supplementary material for: Australian Psychologists Experiences with Digital Mental Health: a Qualitative Investigation
Source: J Technol Behav Sci. 2022 Aug 16:1–11. Online ahead of print. doi: 10.1007/s41347-022-00271-5 (PMC9381152; doi:10.1007/s41347-022-00271-5)
Supplement: Supplementary file 1 — Supplementary file1 (DOCX 27 KB) [file 41347_2022_271_MOESM1_ESM.docx]

**Supplementary Material: Semi-Structured Interview Schedule**

**Interview Questions (with example prompts)**

1. To start, can you please tell me a bit about your work as a psychologist?
2. What motivated you to participate in this study on digital mental health?
3. How would you describe digital mental health?
   1. When you think of digital mental health, what comes to mind?
   2. What resources come to mind when you think of digital mental health?
   3. Are you familiar with research in this area? If yes, what are your thoughts on the research in this area?
   4. Has your view on digital mental health changed at all since COVID-19?
4. Can you please tell me a bit about your experiences incorporating digital mental health into your treatment protocol?
   1. If yes:
      1. How have you used these resources? Which stage of treatment? What type of clients
      2. What type of resources have you used?
      3. What has your experience been like?
      4. What’s important to you when you’re selecting a digital mental health resource?
      5. When and why do you choose to incorporate these resources?
      6. Are there certain clients that these resources are more suitable for?
      7. How would you choose a particular resource?
   2. If no:
      1. Why do you think you haven’t used them?
      2. Would you like to use digital mental health resources as part of treatment?
      3. Do any of your colleagues use digital mental health resources as part of their treatment?
5. From your perspective, what may be some of the benefits associated with digital mental health/integrating it into treatment approach?
6. From your perspective, what may be some of the disadvantages or challenges associated with digital mental health resources?
7. On a broader level, what do you think some of the barriers for digital mental health might be in Australia?
8. What type of things do you think are some of the drivers for digital mental health in Australia?
9. If you could wake up tomorrow and anything was possible, what would you like to see happen in the mental health space?
10. Is there anything that I did not ask you that you would like to talk about or that is important for me to know?
